# Qat Chewing as an Independent Risk Factor for Periodontitis: A Cross-Sectional Study

**DOI:** 10.1155/2013/317640

**Published:** 2013-02-21

**Authors:** Ali Kaid Al-Sharabi, Hussien Shuga-Aldin, Ibrahim Ghandour, Nezar Noor Al-Hebshi

**Affiliations:** ^1^Department of Periodontology, Oral Pathology, Oral Medicine and Radiology, Faculty of Dentistry, University of Sana'a, Sana'a, Yemen; ^2^Department of Periodontology, Faculty of Dentistry, Khartoum University, Khartoum, Sudan; ^3^Department of Preventive Sciences—Periodontology, Faculty of Dentistry, Jazan University, Jazan, Saudi Arabia; ^4^Substance Abuse Research Center (SARC), Jazan University, Jazan, Saudi Arabia

## Abstract

This study assessed the effect of qat chewing on periodontal health, independent of other risk factors. Four hundred qat chewers and 100 nonchewers (20–50 years) were included. Demographic data and detailed information about chewing and smoking were obtained. Periodontal status was assessed using Community Periodontal Index (CPI) and clinical attachment loss (CAL). The qat chewers were older, included more males and smokers, and had worse oral hygiene but higher education levels; the majority were heavy chewers (mean duration of 14.45 years and frequency of 6.10 days/week). Regression analysis identified age, oral hygiene, education level, and cigarette smoking as independent predictors of periodontal destruction. Adjusted for these, qat chewing showed marginally significant association only with CAL (OR = 4.7; *P* = 0.049). The chewing sides showed significantly higher scores than the nonchewing sides; however, equal scores on both sides or lower scores on the chewing sides (possibly no or beneficial effect) were still observed in 50% of the chewers. Heavy qat chewing is shown here as an independent risk factor for attachment loss. However, the possibility that the habit may have beneficial effects in a subset of the chewers cannot be excluded. A holistic model that resolves the existing contradiction is presented.

## 1. Introduction

Qat, or khat *(Catha edulis),* is an evergreen plant belonging to the family Celastraceae which endemically grows in Ethiopia, Kenya, Yemen, Somalia, South Africa, and Madagascar. The fresh leaves and twigs of qat are habitually chewed by millions of local citizens of these as well as neighboring countries due to its stimulating effects attributed to the amphetamine- like stimulant cathinone. The qat chewing habit has spread with immigrants to Europe, North America, and Australia, becoming an international phenomenon [1]. 

Qat is usually chewed in social gatherings as a pastime activity or during special events such as wedding ceremonies. It is also used by drivers, labor, and even students for its reinforcing properties. Typically, 100–200 grams of the fresh leaves and twigs are chewed into a large quid that is retained against the cheek on one side of the mouth; the juice only is swallowed, while the quid is ejected at the end of the chewing session that lasts for 4–10 hours [1]. Although largely viewed as a social habit, long-term heavy chewing has been recently reported to induce a degree of dependence [2].

Qat chewing has been reported to be associated with adverse systemic health effects such as increased risk of cardiovascular events, reproductive problems and psychosis; this has been extensively reviewed in the literature [1, 3–5]. How qat chewing influences oral health has been an active area of research; however, findings have been conflicting with both detrimental and beneficial effects being reported [1]. This controversy has been particularly evident with respect to the effect of the habit on periodontal health as elaborated below.

Rosenzweig and Smith [6] were the first to suggest a possible effect of qat chewing on periodontal tissues. Since then, many attempts to explore this further were made. Several studies did show a significant association between qat chewing and periodontal destruction based on comparisons between chewer and nonchewer groups [7–9]. On the contrary, studies have frequently demonstrated decreased gingival inflammation, decreased pocket depths, or/and lower level of clinical attachment loss on the chewing sides compared to the nonchewing sides [9–12]. Adding to this contradiction, qat chewing has also been repeatedly found to interfere with plaque accumulation and to result in subgingival microbial shifts that are compatible with periodontal health [10, 13–15].

In view of the above, the effect of qat chewing on periodontal health remains unclear. It must be emphasised that most of the previous studies did not properly adjust for the confounding effects of important risk factors including oral hygiene, smoking, age, and history of qat chewing itself. Our hypothesis is that the effect of qat chewing on the periodontium has been overestimated in previous studies because of that. Therefore, the objective of the present study was to assess the effect of qat chewing on periodontal health status among a Yemeni population, independent of other known risk factors.

## 2. Materials and Methods

### 2.1. Sample Recruitment

In this cross-sectional hospital-based study, subjects were recruited among patients attending the dental clinics of Al-thawra Health Institution in Sana'a City (population more than 2 million heterogeneous Yemenis). One hundred qat nonchewers and 400 hundred qat chewers aged 20–50 years were included (the 1 : 4 ratio was based on the estimated 80% prevalence of khat chewing in Yemen). A chewer was defined as a subject who has been chewing at least twice weekly for 5 or more years on only one side of their mouths. Subjects having fewer than 20 teeth and those with history of dipping tobacco use or medical problems were excluded. Verbal consent was obtained from each subject. The study was approved by Khartoum University Higher Education Senate. 

### 2.2. Assessment of Risk Factors

Subjects were interviewed using a structured questionnaire to obtain demographic data and detailed information about oral habits. Data on qat use included duration of the habit in years, frequency of use in days/week, the average period of each session in hours, and intraoral chewing site. Details regarding smoking included type of smoking, duration, frequency, and dose. The state of oral hygiene was visually assessed as good, fair, or poor.

### 2.3. Clinical Outcome Measures

Periodontal status was assessed using the Community Periodontal Index (CPI) and clinical attachment loss (CAL) according to the World Health Organization (WHO) Oral Health Surveys criteria [16]. Briefly, and as recommended, the dentition was divided into sextants and measurements were made around the index teeth in each sextant using a specially calibrated and designed CPI periodontal probe. Each index tooth was given CPI and CAL scores of 0–4 based on pocket depth and clinical attachment loss, respectively (score definitions are provided as footnotes to Tables 3 and 4). The highest score of each parameter was recorded for each sextant. All examinations were performed by a single precalibrated examiner (HMS). 

### 2.4. Statistical Analysis

Firstly, the mean and maximum CPI and CAL scores were calculated for each subject and, among the chewers, for the chewing and nonchewing sides. The data were then summarised as means and standard deviations (for descriptive purposes) and maximum score distribution at the subject-group and chewing/nonchewing side-group levels. Significance of associations between qat chewing and other risk factors as well as between periodontal parameters scores and each of the risk factors was sought using the Chi-square, Mann-Whitney, or Kruskal Wallis tests as appropriate. The Wilcoxon signed-rank test was used for comparisons between the chewing and nonchewing sides. Finally, ordinal logistic regression was used to assess the relation between qat chewing and periodontal parameters adjusting for the effects of age, oral hygiene, smoking, gender, education level, and interaction terms if found. Odds ratios (OR) and 95% confidence interval were calculated. A significance level of 0.05 was considered.

## 3. Results

### 3.1. Sample Description

The demographic characteristics, oral hygiene, and smoking status of the qat chewers and nonchewers are presented in Table 1. The mean age of the chewers (32.48 ± 7.7 y) was significantly higher than that of the nonchewers (29.88 ± 8.4 y). Qat chewing was significantly associated with being a male, having a higher educational level, poorer oral hygiene, and smoking.

### 3.2. Qat Chewing and Smoking History

The chewers reported a duration of qat use of 5–35 years (mean = 14.45 ± 6.77). Threehundred and seventeen (79.3%) of them chewed exclusively on the left side. The chewing frequency was in the range of 2–7 days/week, but the majority chewed qat almost on daily basis (mean = 6.10 ± 1.54 day). The mean chewing session duration was 4.22 ± 1.39 hours (range 1–10). One hundred and seventy-four subjects (34.9%) were smokers, of which 169 (97.1%) were also qat chewers. Cigarette smoking was reported by 135 subjects (77.6%); the rest used water-pipe (locally called mada'a). The mean duration of cigarette smoking was 11.02 ± 6.80 years, and around 95% of cigarette smokers smoked less than 20 cigarettes/day. 

### 3.3. Risk Predictors—Simple Hypothesis Tests

The mean ± SD and maximum CPI and CAL score distribution by age group, gender, oral hygiene, smoking, education level, chewing status, and intraoral site are shown in Tables 2 and 3. Both parameters showed significant association with older age, poor oral hygiene, male gender, and smoking as well as qat chewing (*P* < 0.0001). The chewing sides showed significantly higher scores than the nonchewing sides; however, subject level analysis (Table 4) revealed that the situation is so in only around half of the subjects; no difference between the two sides, or even less destruction on the chewing side, was observed in the rest. 

### 3.4. Risk Predictors: Multivariate Analysis

Putting all variables in multiple ordinal logistic regression models, age, oral hygiene status, education level, and cigarette smoking were identified as the major predictors of both CPI (OR = 1.07, 9.58, 5.46, and 0.036, resp.) and CAL (OR = 1.10, 3.05, 0.036, and 8.75, resp.) (Table 5). Qat chewing status was an additional predictor of CAL (OR = 4.74) with marginal significance (*P* = 0.049).

In exploring for predictors of the different scenarios presented in Table 4, those with equal scores on both sides or with lower scores on the chewing sides were found to be significantly older (*P* = 0.028) and to have significantly more females (*P* = 0.009), longer history of cigarette smoking (*P* = 0.001), worse oral hygiene (*P* = 0.017), and shorter history of qat chewing (*P* = 0.037) compared to those with higher scores on the chewing sides. 

## 4. Discussion

The current study is probably the first to assess the effect of qat chewing on the periodontium, adjusted for the effect of established risk factors, including age, oral hygiene, and smoking. A history of qat use on weekly basis for at least 5 years was used to recruit chewers to ensure adequate exposure to the habit, which is another aspect of the strength of the study. Cross-sectional studies, like the current one, have their known limitations. Nevertheless, they remain widely used in epidemiology for being not expensive and not laborious. In fact, most evidence on the existence of possible associations or risk factors for periodontal diseases comes from cross-sectional studies [17]. The main limitation here, however, is imposed by using CPI. While it is widely used by investigators, this index (previously called CPITN) has been criticised for having severe shortcomings as a screening tool of periodontal status in epidemiological studies [18]. While coupling it with CAL measurement helps solving some of the problems associated with its use, as done in the current study, the index remains inferior to full-mouth recording in estimating the prevalence and extent of periodontitis [19]. Inability to blind the examiner to qat chewing status is another limitation; thus, some sort of measurement bias cannot be excluded. 

This study shows a striking difference between qat chewers and nonchewers in terms of major risk factors of periodontitis, an issue that previous studies have failed to address. Indeed, age, oral hygiene, education level, and cigarette smoking were identified as independent risk factors for periodontitis in this study, which is consistent with evidence from the literature [17]. Prior to statistical adjustment, qat chewing showed strong association with both CPI and CAL scores, also in line with previous reports [7, 8]. However, after adjustment for other factors qat chewing maintained marginally significant association only with CAL but not CPI (i.e., pocket depth) scores, supporting our hypothesis that the effect of qat chewing has been overestimated in previous studies. In fact, Mengel et al. [9] and Yarom et al. [12] did demonstrate that qat chewing was associated with CAL but not pocket depth.

The qat chewing sides in this study showed significantly more periodontal destruction (higher CPI and CAL scores) compared to the nonchewing side, which is not consistent with previous studies in which qat chewing sides were reported to have significantly lower CAL or/and pocket depth scores compared to the opposite sides [9, 11, 12]. However, this cannot necessarily be viewed as contradiction, since subject level analysis revealed that the qat chewing sides had similar periodontal status, or even less destruction, compared to the nonchewing sides in about half of the chewers (no or beneficial effects). To take this further, secondary regression analyses were performed to identify predictors of the different scenarios described in Table 4. It was found that the qat chewing sides were more likely to have worse periodontal status when there was a longer history of qat use, shorter history of cigarette smoking, and better oral hygiene. On the contrary, no or even beneficial effect was likely to happen among less chronic chewers with bad oral hygiene and long history of cigarette smoking. Therefore, the higher destruction observed on the chewing sides in this study is probably because chronic heavy qat chewers (average use for 14.45 years at an average of 6.10 days/week) were overrepresented.

**Figure 1:**
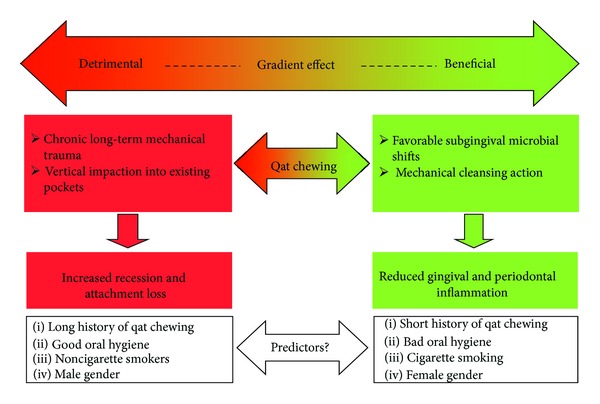
A holistic model for the effect of qat chewing on the periodontium.

In fact, one credible explanation that has not been explored before is that qat chewing probably has both beneficial and detrimental effects and that the nature of overall effect depends on which of the two effects dominate. Based on findings from the current study as well as previous ones, we were able to develop a holistic model for the effect of qat on the periodontium (Figure 1) that resolves contradiction among the studies as elaborated below. 

Qat chewing has been repeatedly shown to modify microbial composition of subgingival biofilm in compatibility with periodontal health [13, 14]. In addition, qat chewing seems to mechanically cleanse dental plaque [10, 15]. In addition, some evidence here is that qat chewing may counteract the destructive effect of cigarette smoking on the periodontium. To this end, qat chewing probably reduces gingivitis and inflammatory periodontitis (pocketing) as shown in some previous studies [10, 12]. On the other hand, heavy qat chewing probably results in chronic trauma and vertical impaction to the periodontium that, on the long term, favors gingival recession and attachment loss, which in turn explains findings from this study as well as a number of previous studies [7, 8].

In conclusion, heavy long-term qat chewing is probably an independent risk factor of clinical attachment loss. The habit, however, seems to have a gradient effect (detrimental, no effect, beneficial) depending on other variables, mainly the status of oral hygiene and history of cigarette smoking. A large-scale, case-control study to explore this further is warranted.

## Figures and Tables

**Table 1:** Demographic data, oral hygiene, education level, and smoking status of both study groups.

	Overall	Qat chewers *n* = 400	Nonchewers *n* = 100	P*
Age group				
20–24 y	99 (19.8%)	62 (15.5%)	37 (37%)	
25–29 y	125 (25.0%)	106 (26.5%)	19 (19%)	
30–34 y	82 (16.4%)	68 (17.0%)	14 (14%)	*<*0.0001
35–39 y	76 (15.2%)	65 (16.2%)	11 (11%)
40–44 y	65 (13.0%)	58 (14.5%)	7 (7%)
45–50 y	53 (10.6%)	41 (10.3%)	12 (12%)	
Gender				
Male	372 (74.4%)	334 (83.5%)	38 (38%)	<0.0001
Female	128 (25.6%)	66 (16.5%)	62 (62%)	
Educational status				
Illiterate	89 (17.8%)	66 (16.5%)	23 (23%)	
Primary	316 (63.2%)	248 (62.0%)	68 (68%)	0.011
High	95 (19.0%)	86 (21.5%)	9 (9%)	
Oral hygiene status				
Good	49 (9.8%)	28 (7.0%)	21 (21%)	
Fair	169 (33.8%)	123 (30.8%)	46 (46%)	<0.0001
Poor	282 (56.4%)	249 (62.2%)	33 (33%)	
Smoking status				
Smokers	174 (34.8%)	169 (42.3%)	5 (5%)	*<*0.0001
Nonsmokers	326 (65.2%)	231 (57.7%)	95 (95%)	

*Chi-square test.

**Table 2:** Descriptive statistics of CPI scores by risk factors.

	Maximum CPI score^#^					
	*N* (%)					
	0	1	2	3	4	Mean, SD
Age group*						
20–24 y	1 (1.0)	18 (18.2)	15 (15.2)	65 (65.6)	0 (0.0)	1.55, 0.55
25–29 y	1 (0.8)	13 (10.4)	14 (11.2)	93 (74.4)	4 (3.2)	1.73, 0.61
30–34 y	0 (0.0)	7 (8.5)	7 (8.5)	67 (81.7)	1 (1.3)	1.84, 0.62
35–39 y	2 (2.6)	3 (3.9)	8 (10.6)	57 (75.0)	6 (7.9)	2.10, 0.72
40–44 y	1 (1.5)	4 (6.2)	3 (4.6)	48 (73.8)	9 (13.8)	2.15, 0.18
45–50 y	0 (0.0)	0 (0.0)	5 (9.4)	42 (79.2)	6 (11.4)	2.23, 0.66
Gender*						
Male	4 (1.1)	22 (5.9)	32 (8.6)	293 (78.8)	21 (5.6)	1.97, 0.67
Female	1 (0.8)	23 (18.0)	20 (15.6)	79 (61.7)	5 (3.9)	1.61, 0.68
Oral hygiene status*						
Good	3 (6.1)	21 (42.8)	4 (8.2)	21 (42.9)	0 (0.0)	1.04, 0.62
Fair	0 (0.0)	21 (12.4)	22 (13.0)	120 (71.0)	6 (3.6)	1.57, 0.46
Poor	2 (0.7)	3 (1.1)	26 (9.2)	231 (81.9)	20 (7.1)	2.21, 0.61
Educational status^NS^						
Illiterate	1 (1.1)	5 (5.6)	6 (7.9)	71 (79.8)	5 (5.6)	1.99, 0.67
Primary	3 (0.9)	32 (10.1)	36 (11.4)	230 (72.8)	15 (4.7)	1.86, 0.71
High	1 (1.1)	8 (8.4)	9 (9.5)	71 (74.7)	6 (6.3)	1.81, 0.66
Smoking status*						
Smokers	2 (1.1)	4 (2.30)	14 (8.0)	142 (81.7)	12 (6.9)	2.04, 0.66
Nonsmokers	3 (0.9)	41 (12.6)	38 (11.7)	230 (70.5)	14 (4.3)	1.78, 0.69
Chewing status*						
Chewers	3 (0.8)	20 (5.0)	29 (7.3)	325 (81.2)	23 (5.7)	1.99, 0.65
Nonchewers	2 (2.0)	25 (25.0)	23 (23.0)	47 (47.0)	3 (3.0)	1.43, 0.69
Intraoral site*						
Chewing sides	5 (1.3)	69 (17.2)	13 (3.3)	299 (74.7)	14 (3.5)	2.33, 0.83
Nonchewing sides	12 (3.0)	154 (38.5)	39 (9.7)	186 (46.5)	9 (2.3)	1.85, 0.90
Overall	5 (1.0)	45 (9.0)	52 (10.4)	372 (74.4)	26 (5.2)	1.87, 0.69

*Significant differences between/among subgroups in CPI maximum score distribution and mean score (*P* < 0.0001); Chi-square, Mann-Whitney, Kruskal Wallis, or Wilcoxon signed-rank tests as appropriate. ^NS^Not significant. ^#^CPI codes—0: healthy; 1: bleeding on probing; 2: calculus detected on probing; 3: pocket depth 4-5 mm; 4: pocket depth 6 mm or more.

**Table 3:** Descriptive statistics of CAL by risk factors.

	Maximum CAL score^#^					
	*N* (%)					
	0	1	2	3	4	Mean, SD
Age group*						
20–24 y	18 (18.2)	68 (68.7)	11 (11.1)	2 (2.0)	0 (0.0)	0.44, 0.35
25–29 y	13 (10.4)	79 (63.2)	27 (21.6)	3 (2.4)	3 (2.4)	0.65, 0.42
30–34 y	2 (2.4)	51 (62.3)	23 (28.0)	5 (6.1)	1 (1.2)	0.81, 0.53
35–39 y	3 (3.9)	33 (43.4)	30 (39.6)	9 (11.8)	1 (1.3)	1.06, 0.57
40–44 y	1 (1.5)	17 (26.2)	29 (44.6)	14 (21.5)	4 (6.2)	1.37, 0.40
45–50 y	0 (0.0)	18 (34.0)	19 (35.8)	15 (28.3)	1 (1.9)	1.26, 0.57
Gender*						
Male	21 (5.6)	188 (50.5)	114 (30.6)	39 (10.5)	10 (2.8)	0.91, 0.60
Female	16 (1.6)	78 (60.9)	25 (19.5)	9 (7.0)	0 (0.0)	0.64, 0.47
Oral hygiene status*						
Good	13 (26.5)	30 (61.3)	5 (10.2)	1 (2.0)	0 (0.0)	0.38, 0.35
Fair	17 (10.1)	108 (63.9)	34 (20.1)	8 (4.7)	2 (1.2)	0.64, 0.42
Poor	7 (2.5)	128 (45.4)	100 (35.5)	39 (13.8)	8 (2.8)	1.05, 0.61
Educational status^‡^						
Illiterate	4 (4.5)	37 (41.6)	35 (39.3)	12 (13.5)	1 (1.1)	0.97, 0.52
Primary	28 (8.9)	177 (56.0)	76 (24.1)	27 (8.5)	8 (2.5)	0.81, 0.61
High	5 (5.3)	52 (54.7)	28 (29.5)	9 (9.5)	1 (1.1)	0.84, 0.53
Smoking status*						
Smokers	5 (2.9)	84 (48.3)	53 (30.4)	27 (15.5)	5 (2.9)	1.02, 0.61
Nonsmokers	32 (9.8)	182 (55.8)	86 (26.4)	21 (6.3)	5 (1.5)	0.75, 0.54
Chewing status*						
Chewers	15 (3.7)	205 (51.3)	126 (31.5)	44 (11.0)	10 (2.5)	0.93, 0.58
Nonchewers	22 (22.0)	61 (61.0)	13 (13.0)	4 (4.0)	0 (0.0)	0.48, 0.43
Intraoral site*						
Chewing sides	32 (8.0)	227 (56.7)	100 (25.0)	35 (8.8%)	6 (1.5%)	1.26, 0.73
Nonchewing sides	91 (22.7)	214 (53.5)	82 (20.5)	11 (2.8%)	2 (0.5%)	0.98, 0.79
Overall	37 (7.4)	266 (53.2)	139 (27.8)	48 (9.6)	10 (2.0)	0.84, 0.58

*Significant differences between/among subgroups in CAL maximum score distribution and mean score (*P* < 0.0001); Chi-square, Mann-Whitney, Kruskal Wallis or Wilcoxon signed-rank test as appropriate. ^‡^Significant difference only in mean scores (*P* = 0.004). ^#^CAL codes: 0: 0–3 mm; 1: 4-5 mm; 2: 6–8 mm; 3: 8–11 mm; 4: 12 mm or more.

**Table 4:** Subject-level comparison of mean CPI and CAL in the qat chewing and nonchewing sides.

	Number (%)
Mean CPI	
In qat chewing side > in qat nonchewing side	211 (52.75%)
In qat chewing side = in qat nonchewing side	124 (31.00%)
In qat chewing side < in qat nonchewing side	65 (16.25%)
Mean CAL	
In qat chewing side > in qat nonchewing side	204 (51.00%)
In qat chewing side = in qat nonchewing side	143 (35.75%)
In qat chewing side < in qat nonchewing side	53 (13.25%)

**Table 5:** Independent predictors of mean CPI and CAL—multiple ordinal logistic regression model^#^.

Predictor	Odds ratio	95% confidence interval	*P*
Mean CPI*			
Age	1.07	1.05–1.10	*<*0.0001
Oral hygiene status	9.58	5.63–16.31	*<*0.0001
Cigarette smoking	5.46	1.13–27.50	0.037
Education level	0.36	0.19–0.70	0.002
Mean CAL**			
Age	1.10	1.07–1.14	*<*0.0001
Oral hygiene status	3.05	2.33–4.00	*<*0.0001
Education level	0.36	0.19–0.70	0.003
Cigarette smoking	8.75	6.37–44.00	0.008
Qat chewing status	4.47	1.07–20.50	0.049

^#^Model assumptions are fulfilled: dependent variable is ordinal; Pearson or/and deviance statistics not significant (model well-fitted).

*Adjusted for gender, water-pipe smoking, qat chewing status, and interaction terms.

**Adjusted for gender, water-pipe smoking, and interaction terms.
